# Enhancing *SHP-1* expression with 5-azacytidine may inhibit STAT3 activation and confer sensitivity in lestaurtinib (CEP-701)-resistant *FLT3*-ITD positive acute myeloid leukemia

**DOI:** 10.1186/s12885-015-1695-x

**Published:** 2015-11-07

**Authors:** Hamid Ali Nagi Al-Jamal, Siti Asmaa Mat Jusoh, Rosline Hassan, Muhammad Farid Johan

**Affiliations:** Department of Haematology, School of Medical Sciences, Universiti Sains Malaysia, 16150 Kubang Kerian, Kelantan Malaysia

**Keywords:** AML, *SHP-1*, CEP-701, Resistance, 5-Azacytidine, STAT3

## Abstract

**Background:**

Tumor-suppressor genes are inactivated by methylation in several cancers including acute myeloid leukemia (AML). Src homology-2 (SH2)-containing protein-tyrosine phosphatase 1 *(SHP-1)* is a negative regulator of the JAK/STAT pathway. Transcriptional silencing of *SHP-1* plays a critical role in the development and progression of cancers through STAT3 activation. 5-Azacytidine (5-Aza) is a DNA methyltransferase inhibitor that causes DNA demethylation resulting in re-expression of silenced *SHP-1*. Lestaurtinib (CEP-701) is a multi-targeted tyrosine kinase inhibitor that potently inhibits *FLT3* tyrosine kinase and induces hematological remission in AML patients harboring the internal tandem duplication of the *FLT3* gene (*FLT3*-ITD). However, the majority of patients in clinical trials developed resistance to CEP-701. Therefore, the aim of this study, was to assess the effect of re-expression of *SHP-1* on sensitivity to CEP-701 in resistant AML cells.

**Methods:**

Resistant cells harboring the *FLT3*-ITD were developed by overexposure of MV4-11 to CEP-701, and the effects of 5-Aza treatment were investigated. Apoptosis and cytotoxicity of CEP-701 were determined using Annexin V and MTS assays, respectively. Gene expression was performed by quantitative real-time PCR. STATs activity was examined by western blotting and the methylation profile of *SHP-1* was studied using MS-PCR and pyrosequencing analysis. Repeated-measures ANOVA and Kruskal–Wallis tests were used for statistical analysis.

**Results:**

The cytotoxic dose of CEP-701 on resistant cells was significantly higher in comparison with parental and MV4-11R-cep + 5-Aza cells (*p* = 0.004). The resistant cells showed a significant higher viability and lower apoptosis compared with other cells (*p* < 0.001). Expression of *SHP-1* was 7-fold higher in MV4-11R-cep + 5-Aza cells compared to parental and resistant cells (*p* = 0.011). STAT3 was activated in resistant cells. Methylation of *SHP-1* was significantly decreased in MV4-11R-cep + 5-Aza cells (*p* = 0.002).

**Conclusions:**

The restoration of *SHP-1* expression induces sensitivity towards CEP-701 and could serve as a target in the treatment of AML. Our findings support the hypothesis that, the tumor-suppressor effect of *SHP-1* is lost due to epigenetic silencing and its re-expression might play an important role in re-inducing sensitivity to TKIs. Thus, *SHP-1* is a plausible candidate for a role in the development of CEP-701 resistance in *FLT3-ITD+* AML patients.

## Background

Acute myeloid leukemia (AML) is a hematological malignancy that occurs as a result of genetic aberrations in hematopoietic progenitor cells [1, 2]. Epigenetic silencing due to DNA hypermethylation is a frequent mechanism of inactivation of tumor suppressor genes (TSG) in variety of human cancers including AML [3]. 5-Azacytidine (5-Aza) is a chemotherapeutic agent that induces DNA demethylation by inhibition of DNA methyltransferase (DNMT) enzymes [4, 5]. The suppression of DNMTs in cancer cell lines induces hypomethylation resulting in decreased viability [6]. CEP-701 is a tyrosine kinase inhibitor (TKI) that potentially inhibits *FLT3* tyrosine kinase and induces hematological remission in patients with AML. However, the majority of AML patients have only moderate and transient responses to tyrosine kinase inhibitors (TKIs) [7–9].

*SHP-1* is a non-transmembrane protein tyrosine phosphatase expressed primarily in hematopoietic stem cells [10–12]. *SHP-1* is a TSG that, in normal cells, negatively regulates Janus kinase/signal transducers and activators of transcription (JAK/STAT) signaling. The loss of *SHP-1* suppressor function results in JAK or STAT activation in cancer cells [13–20]. The JAK/STAT signaling pathway is one of the most important signaling cascades that regulate various cell biological activities including immune response, cell growth, and differentiation [21].

Transcriptional silencing of *SHP-1* due to promoter methylation has been reported in lymphoma and leukemia as well as in many hematopoietic cell lines [12, 22, 23]. Epigenetic silencing of *SHP-1* in myeloproliferative neoplasms and K562 cells results in constitutive activation of JAK/STAT signaling [24]. The restoration of *SHP-1* expression by a demethylating agent such as 5-Aza-2-deoxycytidine (5-Aza2dc) resulted in decreased JAK3, p-JAK3, and p-STAT3 but not STAT3 protein [25]. STAT3 and STAT5 are constitutively activated in myeloid tumors [26]. Resistance to imatinib in chronic myeloid leukemia is conferred by the activation of STAT3 signaling, and the sensitivity is restored by STAT3 inactivation [27].

We hypothesized that JAK/STAT negative regulators may lose their tumor suppression function in TKI-resistant AML cells due to epigenetic silencing, and the re-expression of these genes could re-induce sensitivity to CEP-701. Therefore, gene expression and methylation profiling of *SHP-1* and its downstream targets were studied in *FLT3-ITD* positive AML cells resistant to CEP-701 before and after treatment with 5-Aza.

## Methods

### Lestaurtinib (CEP-701)

CEP-701 was purchased from LC Laboratories (Woburn, MA, USA) and dissolved in DMSO before use. The stock preparation was 1 mM, which was stored at −20 °C according to the manufacturer’s protocol.

### Development of resistant cells

MV4-11, an AML cell line with *FLT3*-ITD, was obtained from the Department of Haematology, Universiti Sains Malaysia (USM), having originally been purchased from American Type Culture Collection (ATCC). The cells were cultured with RPMI 1640 (Life Technologies, Grand Island, NY, USA) supplemented with 10 % fetal bovine serum (FBS; Life Technologies, Grand Island, NY, USA) at a density of 5 **×** 10^4^ cells/mL in a humid incubator with 5 % CO_2_ at 37 °C. A subclone of this line that was resistant to CEP-701, termed MV4-11R-cep, was developed according to the protocol described previously [28]. Briefly, log phase growing MV4-11 cells were co-cultured at a starting dose of 20 nM CEP-701 followed by a step-wise increase in concentration of 10–20 nM for 12 months until the cells were able to survive at the IC_50_ dose of CEP-701 on parental MV4-11. The resistant cell lines were grown in normal medium without CEP-701 for at least 48 h before starting the experiments.

### 5-Azacytidine treatment

5-Azacytidine (5-Aza; Sigma-Aldrich Corp., MO, USA) was dissolved in RPMI-1640 and the stocks at 500 μM were prepared for immediate use or stored at −20 °C, to be used within 2–3 days. Resistant cells were sub-cultured in working solution (5 μM) and incubated in a humidified incubator with 5 % CO_2_ at 37 °C for 4–5 days until confluent. MV4-11R-cep + 5-Aza cells were then sub-cultured in normal medium without treatment for at least one passage before re-treatment with CEP-701.

### Growth inhibition assay

MV4-11 cells were seeded in 96-well culture plates at a density of 1 × 10^4^ viable cells/100 μL/well in triplicates, and were treated with CEP-701. Colorimetric CellTiter 96 AQueous One Solution Cell Proliferation assay (MTS assay; Promega, Madison, WI, USA) was used to determine the cytotoxicity. The IC_50_ values were calculated using GraphPad Prism 3.02 (San Diego, California, USA). Each experiment was performed in triplicate.

### Apoptosis assay

Annexin V–FITC binding assay (BD Pharmingen, San Diego, California, USA) was used as recommended by the manufacturer and analyzed by flow cytometry (BD FACSCanto™, San Jose, California, USA). Analysis was performed with Diva software (FACS Diva, 6.1.2, San Jose, California, USA). Each experiment was performed in triplicate.

### RNA extraction

Total RNA was extracted from MV4-11, MV4-11R-cep, and MV4-11R-cep + 5-Aza cells using the Rneasy® Mini Kit (Qiagen, Valencia, California, USA), the purity and concentration was measured with a NanoDrop ND-1000 spectrophotometer V3.3.0 (NanoDrop Technologies, Berlin, Germany).

### Quantitative reverse transcriptase PCR (RT-qPCR)

High Capacity RNA-to-cDNA kit (Applied Biosystem, Foster City, California, USA) was used to synthesize cDNA according to the manufacturer’s protocol. TaqMan Gene Expression assays (Applied Biosystems) were performed on an Applied Biosystem 7500 Fast Real-Time PCR System according to the manufacturer’s protocol. Glyceraldehyde-3-phosphate dehydrogenase (*GAPDH*) was used as an internal control. ABI 7500 software v2.0.6 (Applied Biosystem) was used to perform relative quantification of 5 target genes, *SHP-1, SOCS-1*, *SOCS-3*, *STAT5a*, and *JAK2* using the comparative threshold cycle (Ct) method.

### DNA extraction

DNA was extracted from MV4-11, MV4-11R-cep, and MV4-11R-cep + 5-Aza cells using the NucleoSpin® Tissue kit (Macherey-Nagel, Düren, Germany) following the manufacturer’s instructions. The concentration and purity of DNA were measured by NanoDrop.

### Methylation-specific polymerase chain reactions (MS-PCR)

One microgram of DNA was treated with bisulfite using the EZ DNA Methylation-Gold™ Kit (Zymo Research, Irvine, NY, USA) according to the manufacturer’s instructions. MS-PCR was performed as described previously [29] and modified DNA was subjected to two separate PCRs. MS-PCR primers were designed to amplify the methylated (M) or unmethylated (U) alleles. *SHP-1* (GeneBank: NM_002831) was amplified using previous designed primers [22]. Universal methylated DNA (Zymo Research, Irvine, NY, USA) was used as a positive control. The 50-μL PCR reaction contained 200 ng of bisulfite-treated DNA, ReddyMix PCR master mix (Bioline Ltd., London, UK) and 0.2 μM of each primer. PCRs were performed in a thermal cycler (PTC-200, Alameda, California, USA). The amplified PCR products were denatured for 2 min at 95 °C followed by 40 cycles: 95 °C for 25 s, 59 °C for 35 s, 52 °C and 72 °C for 65 s, and extension at 72 °C for 5 min. PCR products were electrophoresed on 2 % agarose gels, and visualized by ethidium bromide staining under ultraviolet transillumination. Results from triplicate experiments were used to determine methylation status.

### Pyrosequencing analysis

Twenty microliters (1 μg) of purified DNA from each sample were sent to EpigenDx (Hopkinton, MA, USA) for pyrosequencing analysis. The assay was designed to target six CpG islands in the promoter regions of the *SHP-1* gene.

### Western blot analysis

Protein from MV4-11, MV4-11R-cep, and MV4-11R-cep + 5-Aza cells was extracted by RIPA buffer (Sigma-Aldrich, MO, USA). The three cell lines were incubated with 300 nM CEP-701 for 3 days before protein extraction. BioRad protein dye (BioRad, Hercules, California, USA) and a spectrophotometer (BioPhotometer Plus, Eppendorf, Germany) were employed for the measurement of protein concentrations. Preparation of immunoblotting was performed as described previously [30]. Antibodies used were anti-STAT1, anti-p-STAT1, anti-STAT3, anti-p-STAT3, anti-STAT5, anti-p-STAT5, and anti-β-actin (Thermo Scientific, Waltham, MA, USA).

### Statistical and bioinformatics analysis

Repeated-measures ANOVA and Kruskal–Wallis tests were employed for statistical analyses. All statistical analyses were performed using the SPSS software package (Version 20, SPSS, Armonk, NY, USA) and a *p* value <0.05 was considered as significant.

## Results

### Long-term co-culture of MV4-11 cells with low doses of CEP-701 resulted in resistant cells

**Fig 1:**
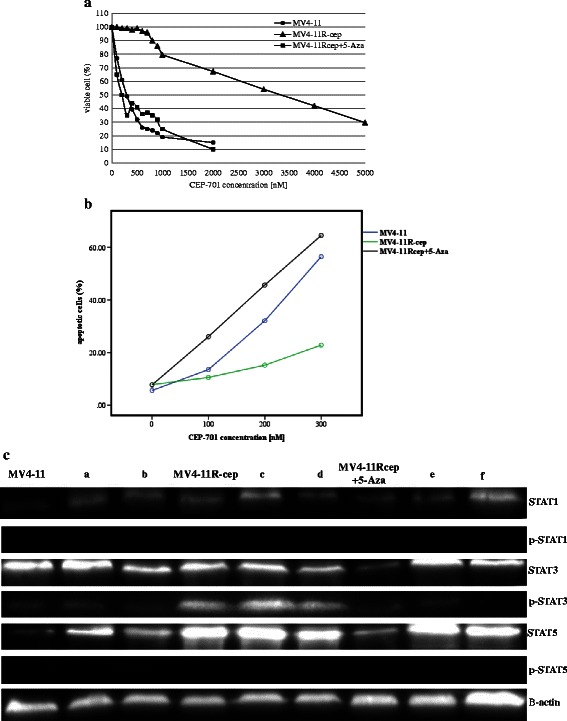
Cell growth inhibition, profile plot of apoptotic cells, and western blot analysis. **a** Cell growth inhibition by CEP-701 in MV4-11 (IC_50_ = 290 nM), MV4-11R-cep (IC_50_ = 3340 nM), and MV4-11R-cep + 5-Aza cells (IC_50_ = 200 nM). The cells were exposed to serial concentrations of CEP-701 for 72 h, and quantified by cell proliferation assay. Each result is presented as the median percentage of proliferation to unexposed control cells. **b** Repeated-measures ANOVA between groups based on concentrations was applied. The profile plot shows the adjusted mean (estimated marginal means) of apoptotic cells for all concentrations of CEP-701 (0, 100, 200, and 300 nM). Although the mean percentages of apoptotic cells before treatment with CEP-701 were almost equal for parental, resistant, and MV4-11R-cep + 5-Aza cells, there was a clear increase in the percentages of apoptotic cells in MV4-11 and MV4-11R-cep + 5-Aza cells with increasing concentration of CEP-701, reaching 58 and 65 %, respectively, at 300 nM. In contrast, the increase of apoptosis in the resistant cells was only 21 % at 300 nM PKC-412 (*p* < 0.001). **c** The phosphorylation of STAT1, STAT3, and STAT5 in MV4-11, MV4-11R-cep, and MV4-11R-cep + 5-Aza cells was assessed by western blotting. STAT3 was activated in MV4-11R-cep cells but not in MV4-11 and MV4-11R-cep + 5-Aza cells. However, STAT1 and STAT5 showed no phosphorylation in all cells; a, b, c, d, e and f indicate other cell lines not included in the present study but they are in agreement with the findings of this study

To verify the resistance of MV4-11R-cep cells to CEP-701, we determined the cytotoxicity and apoptosis of CEP-701 on MV4-11R-cep and parental MV4-11 cells. MV4-11 cells were inhibited by 290 nM CEP-701 whereas the resistant MV4-11R-cep cells were only inhibited by a higher dose (3340 nM). The IC_50_ of CEP-701 on MV4-11R-cep was more than 10-fold higher than that on MV4-11 (*p =* 0.004) (Fig. 1-a). There was a significant decrease in the percentage of apoptotic cells in MV4-11R-cep compared with parental MV4-11 based on incubation of parental and resistant cells in serial concentrations of CEP-701 (*p* < 0.001) (Fig. 1-b).

### Higher sensitivity to CEP-701 in MV4-11R-cep treated with 5-Aza

The MTS assay showed a decrease in the IC_50_ of CEP-701 on MV4-11R-cep + 5-Aza cells compared with that of MV4-11R-cep cells (*p* = 0.011) (Fig. 1-a). The IC_50_ on MV4-11R-cep + 5-Aza was also lower than that of MV4-11 cells, however this difference was not significant (*p =* 0.099).

### Induction of apoptosis in MV4-11 and MV4-11R-cep + 5-Aza in response to CEP-701

**Fig. 2:**
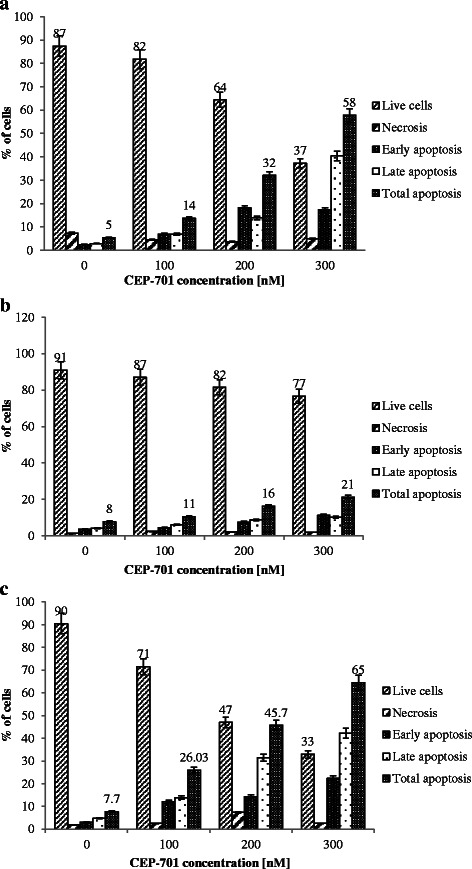
The vitality and fraction of apoptotic cells in MV4-11, MV4-11R-cep, and MV4-11R-cep + 5-Aza cells. Apoptotic cells increased significantly in (**a**) MV4-11 and (**c**) MV4-11R-cep + 5-Aza cells compared with (**b**) MV4-11R-cep cells by increasing concentrations of CEP-701. There was a significant reduction of cell viability from 90% down to 37 and 33% in MV4-11 and MV4-11R-cep + 5-Aza cells, in association with 58 and 65% apoptotic cells, respectively. In contrast, we observed only 21% apoptotic cells in MV4-11R-cep cells at the highest CEP-701 concentration with 77% viable cells remaining (*p* < 0.001)

**Fig. 3:**
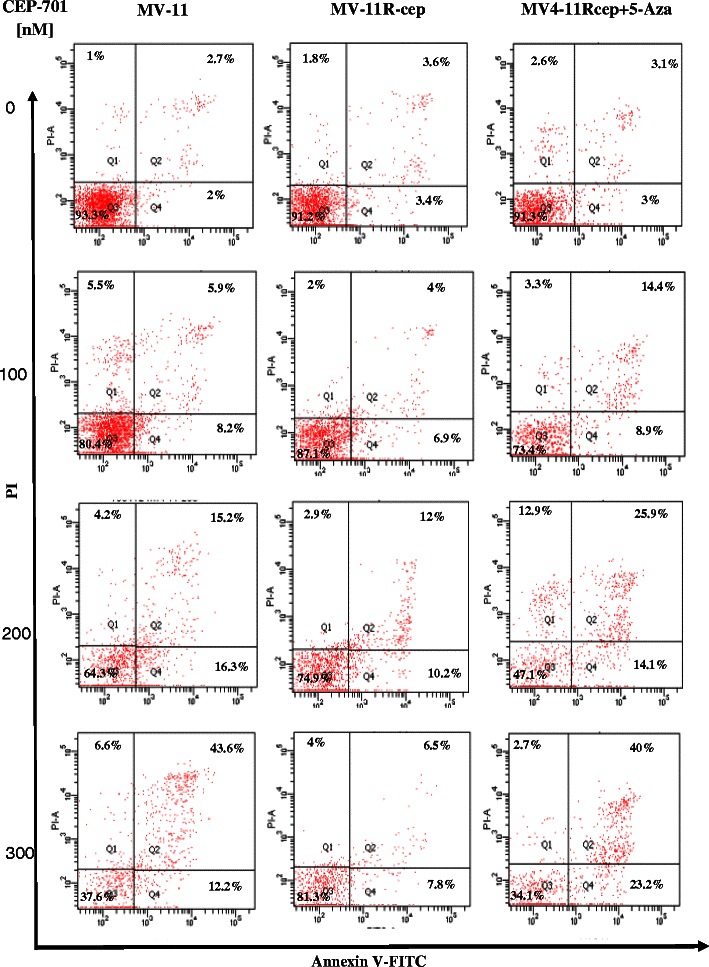
CEP-701 induced decrease of vitality in MV4-11 and MV4-11R-cep + 5-Aza cells. Flow cytometric scatterplots of MV4-11, MV4-11R-cep, and MV4-11R-cep + 5-Aza cells after addition of various concentrations of PKC-412 at 72 h. The data depict the course of apoptotic (Q2 and Q4), live (Q3), and necrotic (Q1) cells. The decrease of vitality induced by CEP-701 was concentration dependent and was greater in MV4-11 and MV4-11R-cep + 5-Aza cells compared with MV4-11R-cep

The vitality and fraction of apoptotic and necrotic cells of MV4-11, MV4-11R-cep, and MV4-11R-cep + 5-Aza cells after various incubations with CEP-701 are shown in Fig. 2. Upon incubation of cells in the presence of 300 nM CEP-701, a significant reduction of cell viability of 90 % down to 37 and 33 % was detected in MV4-11 and MV4-11R-cep + 5-Aza cells, respectively. In contrast, the resistant cell line MV4-11R-cep still showed 77 % viable cells after treatment (Fig. 2). Figure 3 depicts the course of apoptotic (Q2 and Q4) and necrotic (Q1) cells over 72 h measured by Annexin V/FITC-FACS analysis in MV4-11, MV4-11R-cep, and MV4-11R-cep + 5-Aza cells after addition of 100, 200, and 300 nM of CEP-701. The resistant cells showed a significant increase in the viability with obvious decrease in apoptosis after incubation with CEP-701 compared with the parental and MV4-11R-cep + 5-Aza cell lines. Despite the mean percentage of apoptotic cells before incubations with CEP-701 being almost equal for MV4-11, MV4-11R-cep, and MV4-11R-cep + 5-Aza, there was a sharp increase in apoptotic MV4-11 and MV4-11R-cep + 5-Aza cells with increased drug concentration, reaching 58 and 65 % apoptosis, respectively, at 300 nM (Fig. 1-b). In contrast, there was no significant increase in the apoptotic cells in MV4-11R-cep with increased drug concentrations, with only 21 % apoptotic cells at 300 nM (*p* < 0.001).

### Activated STAT3 in resistant acute myeloid leukemia cells

The activation status of STAT1, STAT3, and STAT5 proteins were studied using western blot. Although these proteins were expressed in all cell lines, STAT3 was only activated in the resistant cells and not the parental and MV4-11R-cep + 5-Aza cells. In contrast, there was no differences in the activity of STAT1 and STAT5 between resistant and parental or MV4-11R-cep + 5-Aza cells (Fig. 1-c).

### Restoration of SHP-1 gene expression in MV4-11R-cep + 5-Aza cells

**Fig 4:**
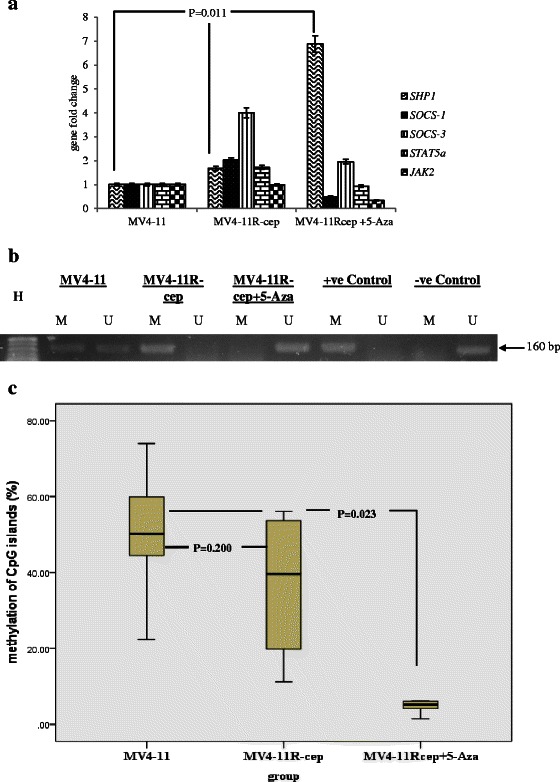
Real-time quantitative PCR (RQ-PCR) and methylation status of *SHP-1* in all cells. **a** The relative normalized ratio of RQ-PCR revealed that, *SHP-1* is re-expressed in MV4-11R-cep + 5-Aza cells 7-fold higher compared with that in MV4-11 and MV4-11R-cep cells (*p* = 0.011). **b** Methylation-specific polymerase chain reaction (MS-PCR) showed methylation of *SHP-1* in MV4-11 and MV4-11R-cep cells but not in MV4-11R-cep + 5-Aza cells. **c** Pyrosequencing analysis revealed low methylation levels of the CpG islands in the promoter region of *SHP-1* in MV4-11R-cep + 5-Aza cells. The Kruskal–Wallis test was applied followed by the Multiple Mann–Whitney Test with Bonferroni correction. The box blot showed a significant lower (*p =* 0.023) of methylation in the CpG islands of *SHP-1* gene in MV4-11R-cep + 5-Aza cells compared with that in MV4-11 and MV4-11R-cep cells. However, there was no significant difference in the methylation levels of CpG islands in the same region of *SHP-1* genes between MV4-11 and MV4-11R-cep cells (*p =* 0.200)

To investigate the correlation between re-expression of *SHP-1* and demethylation, gene expression by RT-qPCR was performed on MV4-11, MV4-11R-cep, and MV4-11R-cep + 5-Aza cells. The results showed a significant up-regulation of *SHP-1* in MV4-11R-cep + 5-Aza cells compared with MV4-11 and MV4-11R-cep cells (*p* = 0.011 and *p* = 0.002, respectively; Fig. 4-a).

### Low methylation of SHP-1 gene in MV4-11R-cep + 5-Aza cells

To relate the expression of *SHP-1* with methylation status, MS-PCR and pyrosequencing analysis were performed on DNA from the three cell lines as well as positive and negative controls. MS-PCR revealed methylated *SHP-1* in parental MV4-11 and resistant MV4-11R-cep cells but not MV4-11R-cep + 5-Aza cells (Fig. 4-b). The methylation status was confirmed by pyrosequencing analysis, which revealed a significantly lower level of methylation in CpG islands in the promoter region of the *SHP-1* gene when treated with 5-Aza compared with untreated MV4-11 and MV4-11R-cep cells (*p* = 0.023). However, there was no significant difference in the methylation of CpG islands in the promoter region of *SHP-1* in MV4-11 cells compared with MV4-11R-cep cells (*p* = 0.200; Table 1 and Fig. 4-c).

**Table 1:** Percentage of methylation of CpG islands in the promoter region of the *SHP-1* gene

Sample ID	CpG-11	CpG-10	CpG-9	CpG-8	CpG-7	CpG-6	Mean	Min	Max
MV4-11	22.4	48.8	74.0	59.9	51.6	44.5	50.2	22.4	74.0
MV4-11R-cep	11.2	19.9	56.1	53.7	41.5	37.9	36.7	11.2	56.1
MV4-11R-cep + 5-Aza	4.9	4.2	6.1	6.2	5.5	1.5	4.7	1.5	6.2
Low Meth Control	6.8	6.9	2.7	10.5	8.3	5.9	6.8	2.7	10.5
Med Meth Control	52.2	53.0	52.1	42.7	48.4	49.1	49.6	42.7	53.0
High Meth Control	93.7	94.0	92.5	74.5	83.3	93.8	88.6	74.5	94.0

Pyrosequencing analysis showing methylation levels of six CpG islands of the *SHP-1* gene. A significant higher level of methylation was observed in the CpG islands of *SHP-1* in MV4-11 and MV4-11R-cep compared with in MV4-11R-cep + 5-Aza cells (*p =* 0.023), using the EpigenDx kit (Hopkinton, MA, USA)

## Discussion

Resistance to TKIs remains a challenge in the treatment of AML patients. The mechanism of acquired resistance to TKIs has been studied *in vitro* and *in vivo* [7, 31–35] but is still not fully understood. Aberrant methylation of tumor suppressor genes (TSG) such as *SOCS-1*, *SOCS-3*, *SHP-1*, and *PRG2* has been documented in a variety of cancers including hematological malignancies [3, 36–42], and is a plausible means by which cells can acquire therapy resistance.

In the present study, *FLT3*-ITD+ AML cells resistant to CEP-701 were developed by overexposure of parental cells to the drug. Acquired resistance was confirmed by cytotoxicity and apoptosis assays that showed significant differences in parental compared with resistant cells. The IC_50_ of CEP-701 on resistant MV-11R-cep was more than 10-fold higher than that of parental MV4-11 cells (*p* = 0.004) and acquired resistance was associated with low apoptosis (*p* < 0.001). This is in agreement with previously reported studies on the development of resistant cell lines to ABT-869 and PKC-412 [7, 31].

Gene expression analysis revealed low expression of *SHP-1* and *PRG2* in MV4-11 and MV4-11R-cep cells (data not shown for *PRG2* gene). However, after treatment of MV4-11R-cep cells with 5-Aza, we observed re-expression of *SHP-1* and *PRG2* that was associated with inhibition of STAT3 activity. Moreover, the transcriptional silencing of *SHP-1* and *PRG2* genes was due to hyermethylation of CpG islands in the promoter regions of both genes in MV4-11 and MV4-11R-cep cells. Transcriptional silencing of TSGs is mediated by DNMTs in tumor cells [43–46]. The gene expression analysis also showed significant up-regulation of *DNMT1*, *DNMT3a*, and *DNMT3b* (data not shown), which have been reported to regulate the expression of TSGs through methylation of CpG islands in the promoter regions [47, 48]. Methylation profiling revealed hypermethylation of CpG islands in the promoter regions of *SHP-1* and *PRG2* in MV4-11 and MV4-11R-cep cells (data not shown for *PRG2* gene). These findings suggest that, the up-regulated DNMTs in the parental and resistant cells methylate the CpG islands of *SHP-1* and *PRG2* genes, resulting in their transcriptional silencing. Our findings are consistent with previous reports that revealed hypermethylation of *SHP-1* and *PRG2* in leukemic cell lines [22, 38, 42, 49].

In hematological malignancies and leukemic cell lines, the tumor-suppressing function of *SHP-1* is lost because of promoter methylation, resulting in constitutive activation of JAK/STAT signaling [22, 24, 39, 50, 51]. Epigenetic silencing of one of the JAK/STAT negative regulators is sufficient for activation of STAT signaling [40]. Methylation of *SHP1* is involved in the constitutive activation of STAT3 [50], and a low level of *SHP-1* is not sufficient to inhibit activated STAT3 [25]. Transcriptional silencing of *SHP-1* also plays a role in the development of resistance to imatinib in *BCR-ABL1*-positive CML cells [51]. Similarly, constitutive activation of STAT3 and STAT5 are common events in myeloid leukemia and have previously been implicated in resistance to TKIs [7, 26, 52]. Bewry, et al. [27] suggested that, the activation of STAT3 is an important mechanism of imatinib resistance. Likewise, overexpression of *PRG2* in myeloid cells blocked G-CSF-dependent proliferation and increased apoptosis [53]. However, epigenetic silencing of *PRG2* is associated with higher proliferation and lowered apoptosis in pancreatic cancer cells [54] and leukemic cells [42].

In the present study, we observed activation of STAT3 in MV4-11R-cep cells but not in MV4-11 cells. The findings suggest that, activated STAT3 could be involved in the acquisition of resistance to CEP-701 in MV4-11R-cep cells, which is consistent with previous reports in which, the activation of STAT3 was associated with acquired resistance to ABT-869 in AML [7], and to imatinib in CML [27, 51]. After treatment of MV4-11R-cep with 5-Aza, STAT3 was inactivated and cells showed higher sensitivity to CEP-701. This finding is in accordance with other reports [7, 25, 51]. Our data suggest a crucial role for STAT3 in the development of resistance to TKIs and inhibition of STAT3 phosphorylation provides an effective means of re-inducing sensitivity.

STAT3 is negatively regulated by TSGs such as *SHP-1, SOCS-1*, and *SOCS-3* [7, 41, 51, 55, 56]. Re-expression of these genes by 5-Aza or 5-Aza2dc results in inactivation of STAT3 [7, 25, 51, 57, 58]. In addition, inactivation of STAT3 enhances apoptosis and restores sensitivity towards TKIs [27, 59]. Similarly, we found that the re-expression of *SHP-1* and *PRG2* is associated with inactivation of STAT3 in 5-Aza-treated cells. Moreover, the sensitivity of MV4-11 and MV4-11R-cep + 5-Aza cells towards CEP-701 was significantly higher with low IC_50_, at only 200 nM compared with 3340 nM in MV4-11R-cep (*p* = 0.011). The findings suggest that, the restoration of expression of *SHP-1* and *PRG2* could induce sensitivity towards CEP-701 through inactivation of STAT3. Our observations are supported by previous reports [7, 25, 27, 31, 51, 59].

We also found that, increasing CEP-701 concentration caused a significant increase of apoptosis in 5-Aza-treated resistant cells compared with the untreated resistant cells (*p* < 0.001) In addition, we observed no significant difference when CEP-701 concentration was increased by 100 nM in untreated resistant cells whereas the 5-Aza-treated resistant cells showed significant differences at each incremental increase in TKI concentration. Taken together, our results indicate that, re-expression of *SHP-1* and suppression of STAT3 are associated with induction of apoptosis in TKI-resistant *FLT3*-ITD+ AML cells. These findings are similar to those previously reported [7, 60–62]. Enhanced re-expression of *SHP-1* could therefore play role in the management of CEP-701-resistant patients. Further studies are needed to clarify the correlation between the re-expression of the *SHP-1* gene with STAT3 inhibition and to confirm the clinical utility of this approach.

## Conclusion

The epigenetic silencing of *SHP-1* results in loss of its tumor suppressor function and re-expression of *SHP-1* by 5-Aza may enhance sensitivity to CEP-701 through inactivation of STAT3.
